# The Role of Thiamine and Effects of Deficiency in Dogs and Cats

**DOI:** 10.3390/vetsci4040059

**Published:** 2017-11-24

**Authors:** Georgia Kritikos, Jacqueline M. Parr, Adronie Verbrugghe

**Affiliations:** Department of Clinical Studies, Ontario Veterinary College, University of Guelph, 50 Stone Rd. E., Guelph, ON N1G 2W1, Canada; gkritiko@uoguelph.ca (G.K.); parr@uoguelph.ca (J.M.P.)

**Keywords:** vitamin B_1_, water-soluble vitamins, dog food, cat food, nutrient imbalance

## Abstract

Recent pet food recalls for insufficient dietary thiamine have highlighted the importance of adequate thiamine intake in dogs and cats, as thiamine is an essential dietary nutrient with a critical role in energy metabolism. Prolonged thiamine deficiency leads to clinical signs that can span several organ systems, and deficiency can be fatal if not reversed. In this review, the current knowledge of thiamine metabolism will be summarized. Dietary recommendations for dogs and cats will be discussed, and the risk factors and clinical signs associated with thiamine deficiency will be examined.

## 1. Introduction

Thiamine (vitamin B_1_) is a water-soluble vitamin and an essential dietary nutrient in dogs and cats. When in the form of thiamine diphosphate (TDP), it has a critical role as a cofactor in carbohydrate metabolism, in the production of nucleotides and of nicotinamide adenine dinucleotide (NADH), and for nervous system function. Other forms of thiamine include unphosphorylated (free) thiamine, and thiamine with one or three phosphate groups (thiamine monophosphate and triphosphate, respectively). These forms have not been as well-studied, but may be important for nervous system function and may have additional functions that are not currently known [1,2,3]. Thiamine deficiency has been reported in both dogs [4,5,6] and cats [6,7,8,9] and recent recalls of thiamine-deficient cat foods have shown that this still continues to be a clinically relevant disease. Still, as the majority of research has been done in species other than dogs and cats, further investigation of species-specific variations of thiamine metabolism and requirements is warranted.

## 2. Dietary Thiamine Requirements in Dogs and Cats

Dogs and cats cannot synthesize thiamine, and therefore require thiamine as part of their diet [10]. According to nutritional requirements determined by the National Research Council (NRC), cats require approximately two to four times more thiamine per day than dogs [10]. This is based on research where thiamine deficiency was induced through feeding of a thiamine-deficient diet. Underlying differences in metabolism between cats and dogs are currently unknown.

Dietary thiamine in dog and cat foods can be supplied in raw ingredients or can be added as a synthetic supplement in the form of thiamine mononitrate or thiamine hydrochloride [10,11]. Presently, dietary thiamine recommendations have been determined for dogs and cats from case reports and studies measuring varying rates of thiamine intake and resultant health status in dogs and cats. In North America, many commercial pet food manufacturers follow Association of American Feed Control Officials (AAFCO) guidelines when formulating diets, which historically are based on recommendations for adequate intake (AI) and recommended allowance (RA) by the NRC. Currently, due to lack of data, there is no known safe upper limit (SUL) for thiamine [12]. Recommendations for thiamine concentrations of adult dog and cat diets were updated by AAFCO in 2016 to match the values recommended by the NRC [13]. In Europe, the Fédération Européenne de l’Industrie des Aliments Pour Animaux Familiers (FEDIAF; European Pet Food Industry Federation) has its own set of recommendations for nutrient intake in dogs and cats, which are based on the NRC’s AI recommendations for adult dogs and cats [14]. The recommendations for dietary thiamine in adult dogs and cats according to the NRC, AAFCO, and FEDIAF are summarized in Table 1.

## 3. Thiamine Status in Healthy Dogs and Cats

Several methods exist in veterinary and/or human medicine to determine thiamine status. Some are indirect methods that assess thiamine status, such as by measuring the activity of enzymes for which thiamine is a cofactor, while others directly quantify concentrations of thiamine or its phosphate esters.

### 3.1. Indirect Methods to Assess Thiamine Status

Thiamine status can be estimated using functional tests that measure the activity of enzymes for which thiamine is a cofactor to determine thiamine status. These tests include erythrocyte transketolase activity (ETKA) and the increase in transketolase (TK) activation that is measured after addition of TDP in vitro, also called TDP effect [15,16,17,18]. There have been few reports in veterinary literature of indirect thiamine status assessment in blood or tissues of dogs [19] and cats [20]. Moreover, these tests are not without limitations, as results may be impacted by various physiological and technical factors [21,22,23,24].

Thiamine diphosphate is a cofactor for pyruvate dehydrogenase, which converts pyruvate to acetyl co-enzyme A (acetyl CoA) as part of aerobic metabolism. In the absence of adequate enzyme, pyruvate is instead converted to lactate via lactic acidosis (i.e., anaerobic metabolism) [17]. In human medicine, thiamine deficiency has been suspected based on the presence of elevated blood lactate concentrations [25,26]. Although blood lactate analysis is performed routinely in dogs and cats [27], the link with thiamine status has not been investigated. Nonetheless, high urinary concentrations of lactic acid have been linked with thiamine deficiency in dogs and cats [28,29]. Still, elevated lactate concentrations in blood and urine are not specific to thiamine deficiency as hypoxic and non-hypoxic lactic acidosis occurs with many disorders [27]. Simultaneous assessment of both lactate and pyruvate concentrations may be a better predictor of thiamine status [30].

Thiamine status can also be inferred from assessment of urinary thiamine concentrations, where urinary thiamine concentrations are measured per unit of creatinine to account for renal function [31,32,33]. A significant correlation is present between thiamine intake and urinary thiamine excretion in adult humans, however, excretion rates can vary widely between individuals [33]. Analysis of urinary thiamine requires more evaluation before it can be used as a method to assess thiamine status in dogs and cats.

Measurement of the growth response of microbial or protozoal species (e.g., yeast, algae) that require thiamine have previously been used to assess whole blood thiamine status in dogs and cats [34,35]. However, microbial methods have not been validated in dogs and cats. These methods are also subject to limitations and are currently not commonly used for thiamine analysis [36].

### 3.2. Direct Methods to Assess Thiamine Status

Currently, the first choice method of thiamine analysis is to use high-performance liquid chromatography (HPLC) to determine TDP concentrations in red blood cells [37]. This method, validated in humans [37] and often used in canine and feline studies [38,39,40], is preferable as it directly measures thiamine concentrations in blood and is therefore not impacted by the physiological processes that can impact results of indirect analyses. Additionally, TDP is stable in whole blood for up to 48 h at room temperature, and hemolysates and whole blood are stable at −70 °C for at least 7 months [37]. Thiamine concentrations in red blood cells obtained by HPLC are correlated with concentrations in whole blood [37], and are also correlated with results of TK activation assays and TDP effect [37,41].

### 3.3. Thiamine Reference Range in Dogs and Cats

Little is known about blood thiamine status in dogs and cats. Thus, more research is required to determine an accurate reference range for thiamine in both species.

A small study that compared whole blood thiamine status in one thiamine-deficient dog and six control dogs reported lower thiamine status in the thiamine-deficient animal compared to controls, with control dogs having a mean blood thiamine concentration of 95 µg/L (range: 84–104 µg/L) [35]. However, diets were not nutritionally balanced, and small sample sizes mean that results cannot be generalized to the canine population. Another study determined whole blood thiamine concentrations in 25 dogs and 29 cats and also used microbial methods, and found a median blood thiamine concentration of 72 µg/L (range: 46–112 µg/L) in dogs, and a median of 48 µg/L (range: 20–90 µg/L) in cats [34]. However, there were methodological concerns in this study. Dogs and cats in this study included growing animals, adults, and senior animals. In addition, how the animals’ health status was determined was not clarified and dietary intake was not assessed. Both of the above studies used protozoal methods to assess thiamine status by measuring metabolically active forms of vitamins, and while the authors of the second study stated that the method had been validated in humans and rats, it was not validated in dogs and cats and is not a method that is used with any regularity.

In cats, a recent case series used HPLC to analyze TDP status in whole blood of three healthy animals and compared this to the thiamine status of cats diagnosed with thiamine deficiency. The authors found blood TDP concentrations of 47.0 ± 7.8 µg/L in the healthy cats [38]. The TDP status of whole blood in 64 adult dogs has also been determined using HPLC. Dogs had a mean ± SD baseline blood TDP concentration of 89.7 ± 20.7 µg/L, which represented a thiamine status of dogs being fed a variety of diets [39]. The authors later put the dogs on one of four diets that each contained known thiamine contents for four months, and found that thiamine status did not differ significantly between these diets, nor did it differ from the baseline value for any of the diets. However, thiamine concentrations were not adjusted based on hematocrits when conducting thiamine analyses. This could have affected the observed results, as TDP in the blood is predominantly found in red blood cells [42], and thus any fluctuations in hematocrit could affect resultant blood thiamine concentrations.

More recently, an assessment of RBC thiamine concentrations in 22 dogs and eight cats was conducted using HPLC [40]. Blood thiamine concentrations were adjusted based on animals’ hematocrits. All dogs and cats had been put on one of three complete and balanced canine or feline diets, respectively. Mean ± SD TDP concentrations for dogs and cats were a respective 311.4 ± 46.8 and 868.2 ± 344.0 µg/L. Unphosphorylated thiamine (free thiamine) status of dogs was reported to be 350.3 ± 50.3 µg/L, but was not assessed in cats [40]. The reason for the higher TDP concentrations observed in this study in comparison to previous studies is unknown, but may have been related to differences in methodology compared to previous studies [40]. Additionally, in blood cells, thiamine diphosphate is typically the predominant form, while free thiamine makes up a much smaller proportion [17], which is in contrast with the findings of this study. However, this study was the first to assess free thiamine status in dogs. Additional research is required to determine a reference range for TDP and free thiamine in dogs and cats.

## 4. Thiamine Deficiency in Dogs and Cats

### 4.1. Prevalence of Thiamine Deficiency

The prevalence of thiamine deficiency in dogs and cats, both healthy animals and patients with disease, is currently unknown.

However, more information is available from human studies that typically examine the prevalence of thiamine deficiency in specific diseases, by utilizing indirect methods (ETKA and TDP effect) or direct methods (HPLC) to assess thiamine status. When compared to the thiamine status of hospitalized control subjects, the prevalence of poor thiamine status has been found to be elevated in individuals with diabetes mellitus (type 1 and 2) [43], congestive heart failure [32], and chronic alcoholism [44]. However, comorbidities, use of certain medications, diet or nutritional status, and age can impact thiamine status and should be considered as potential factors influencing results in these populations. Thiamine deficiency in elderly people in the UK has been reported to occur in 8–31% of non-institutionalized individuals and between 23–40% in individuals living in nursing homes [45]. One study found that 10% of human intensive care unit (ICU) inpatients with septicemia are thiamine deficient upon presentation, which increased to 20% within 72 h of admission [46]. Thiamine deficiency has been reported in 33–91% of hospital patients with heart disease [47]. Overall, approximately 20% of adult ICU inpatients are thiamine deficient upon admission [48].

### 4.2. Risk Factors and Etiology of Thiamine Deficiency

#### 4.2.1. Inadequate Dietary Thiamine Intake

Thiamine is an essential dietary nutrient and is not sequestered in large quantities in the body, with excess dietary thiamine being excreted in urine [10]. Consistent thiamine intake is therefore critical to prevent deficiency.

a. Unconventional and Alternative Diets

Unconventional and alternative diets include cooked or raw homemade diets, as well as commercially-produced diets that resemble homemade diets or that require some preparation from owners (e.g., addition of water or meat) [49]. These types of diets for pets are a growing trend in North America, as owner concerns or suspicions about the nutritional quality of commercial pet foods increase [50].

The majority of these diets are not nutritionally adequate [49,51,52]. There have been reports of dogs and cats developing signs of thiamine deficiency as a result of being fed unbalanced homemade cooked diets [4,8,35]. Nutritional analyses have shown that homemade diets tend to lack essential macro- and micro-nutrients, including thiamine, when they are intended for adult maintenance [51,53]. Insufficient thiamine has also been found in homemade diets intended for the treatment or management of specific diseases [54,55]. Additionally, nutrient imbalances have been seen in homemade and commercial raw diets, though thiamine content has not specifically been assessed in these diets [52,56,57].

Canine diets formulated by both veterinarians and non-veterinarians often do not meet nutritional requirements for dogs [51]. Thus, homemade diets should be formulated by board-certified veterinary nutritionists to ensure nutritional adequacy. Even when homemade diets have been formulated to meet nutritional adequacy recommendations, there may be variability in the exact nutritional composition of the ingredients [58]. This makes it difficult to keep the nutrient content of homemade diets consistent between batches. There are also concerns with owners not closely following the recipe, as owners may switch ingredients or alter the preparatory methods of the recipe without consulting a veterinary nutritionist, which can lead to unbalanced diets [58,59].

b. Conventional Pet Food 

In addition to homemade diets, conventional dry and wet diets may contain inadequate thiamine concentrations.

In the United States and Australia, over 90% of dogs receive conventional dry or wet dog foods as at least 50% of their diet [60,61]. Also, cat owners have been found to be more likely to feed conventional pet foods than dog owners [61]. Conventional cat foods make up at least 50% of the diet for more than 98% of cats [60,61]. Since dogs and cats tend to receive most of their nutrition from conventional diets, it is imperative that these diets contain adequate concentrations of essential dietary nutrients, including thiamine.

Nonetheless, diets intended for intermittent or supplemental feeding only may provide inadequate thiamine and subsequently lead to development of signs of thiamine deficiency [38]. Thus, feeding conventional diets that follow AAFCO or FEDIAF recommendations and that contain a statement on their label declaring that they are complete and balanced for the appropriate species and life stage, minimizes the risk of poor dietary thiamine intake in dogs and cats.

Still, even with complete and balanced conventional pet foods, the risk for thiamine deficiency may exist. Thiamine is very labile and readily degrades under different conditions, including exposure to oxygen, heat, and neutral and alkaline pH [62,63,64,65,66]. This can pose problems for ensuring that processed diets meet requirements for thiamine concentrations. In pelleted and extruded ruminant feed, thiamine hydrochloride and mononitrate degradation is approximately 4.0% and 4.5% per month, respectively [67]. Cooking of thiamine-containing foods has variable losses of thiamine, as the exact cooking procedure will lead to different percentages of loss of thiamine [66]. The effect of freezing on thiamine retention is currently unknown. Studies typically examine freezing in addition to another form of processing (e.g., cooking, blanching, canning) [68,69,70,71], making results difficult to interpret. One study found that frozen spinach contained higher thiamine content after storage compared to baseline values, but the authors were unable to determine a reason for this result [72].

Pet food manufacturers may compensate for anticipated thiamine degradation by adding additional synthetic thiamine prior to cooking (i.e., extrusion or baking for dry food, and canning or cooking for wet food). Despite this, the final products may not contain adequate quantities of thiamine. Differences in manufacturing procedures between factories and between specific diets may differentially affect thiamine losses, making it difficult to estimate general losses across all dry or wet diets. In general, higher cooking temperatures during extrusion can lead to greater losses of thiamine compared to lower cooking temperatures [67,73]. However, a recent review examining effects of extrusion on dry pet foods also notes that while higher temperatures may lead to greater thiamine losses, different retention times during extrusion can also lead to variability in thiamine losses between diets [74].

Wet foods seem to be especially susceptible to thiamine deficiency, based on pet food recall information. Since 2010, six recalls of commercial cat foods have occurred for suspected or confirmed thiamine deficiency in North America. These diets all had AAFCO nutritional adequacy claims. Recalls consisted of 17 commercial canned cat foods, compared to five extruded dry cat foods and one raw cat food [75]. The Food Standards Agency (FSA) in the United Kingdom has also reported a recent thiamine-related recall for three dry extruded cat foods [76], and more recently, there has been a recall of eight canned cat foods in Australia that have been found to contain insufficient thiamine [77]. A recent study examining thiamine concentrations in samples of 90 commercial canned cat foods in the United States, all of which claimed to meet AAFCO guidelines, found that 12 of these foods (13.3%) were thiamine-deficient compared to AAFCO (2014) recommended concentrations, and 14 foods (15.6%) were deficient compared to the slightly higher NRC recommended concentrations [78]. With the 2016 increase in AAFCO recommended concentrations to match those of the NRC, the number of diets in this study that were deficient according to AAFCO recommendations would have been comparable to the NRC (15.6%). The authors of this study also found that paté foods and foods produced by small manufacturing companies had a higher proportion of low thiamine concentrations than non-paté foods and foods produced by large manufacturing companies, respectively. The authors speculated that these differences may have been due to thiamine degradation as a result of the extra processing required to produce paté foods, as well as the ability of larger manufacturers to exert better quality control and conduct more frequent testing for thiamine. The cross-sectional nature of this study, as well as the possibility of inter-batch variation between products and the absence of random selection from all commercially available products, may have impacted results. Additionally, a study that reported thiamine retention after 18 months of storage found 0% thiamine losses in canned dog and cat food over 18 months of storage, but reported storage-related losses of 34.2% and 57.5% in extruded dry dog and cat food, respectively [79]. However, the original research could not be consulted as this research is no longer archived at its publisher, and storage conditions of the diets are therefore unknown.

c. Presence of Anti-Thiamine Compounds 

Anti-thiamine compounds or thiamine agonists are synthetic or natural compounds that inactivate thiamine by changing thiamine’s chemical structure.

Thiamine is unique in that it is the sole vitamin for which an enzyme has evolved that can destroy it. This enzyme, thiaminase, is present in the flesh of dead fish and in some shellfish and bacterial species. Thiaminases cleave thiamine to make it biologically inactive [66,80,81]. Thiamine deficiency has been documented in animals that have been fed diets containing predominantly raw fish, including dogs and foxes [4,82]. Thiaminases present in fish are denatured by high heat, with varying sensitivity [83,84]. Therefore, the risk for thiamine deficiency may be present in both raw and inadequately cooked fish-based diets for dogs and cats. These diets may benefit from adding additional supplemental thiamine during processing or as an oral supplement during feeding.

Additionally, the use of sulfites such as sulfur dioxide to preserve meats has been implicated in thiamine loss in foods. There have been documented cases of dogs and cats developing signs of thiamine deficiency after eating sulfite-preserved meat as a primary component of their diet [5,6,9]. This is likely due to sulfur’s actions in converting thiamine to thiamine disulfide, as this form of thiamine has poor bioavailability in the body [66]. This thiamine-destroying effect occurs in non-meat products, such as parboiled rice, as well [85]. In commercial pet foods, the use of sulfur as a preservative could lead to the loss of bioavailable thiamine and subsequent thiamine deficiency in pets when fed these commercial diets as the primary source of nutrition. Presently, sulfites are not permitted in foods containing meat or in sources of thiamine by the United States Food and Drug Administration [86], and AAFCO has similar restrictions on the usage of sulfur and sulfites [13]. This decreases the likelihood of thiamine deficiency caused by sulfites in pets in North America.

Plant-derived anti-thiamine factors, heat-stable compounds known as polyhydroxyphenols, which include caffeic acid, phenols, flavonoids, and tannins, are present in certain plants and destroy thiamine by an oxidative process that transforms it to non-absorbable thiamine disulfide [17,63,66,87,88,89,90]. Plants containing polyhydroxyphenols include coffee, tea, and some fruits and vegetables, such as blueberries and red cabbage [17]. While research in dogs and cats has not been conducted to determine the effect of plant-derived anti-thiamine factors on blood nutrient concentrations, the presence of anti-thiamine factors in plant matter may be of importance for dogs and cats being fed homemade diets or large portions of table scraps containing ingredients with these compounds.

d. Interactions with Other Nutrients

Little has been documented about the interactions of thiamine with other nutrients. A study in kittens showed that high concentrations of dietary glutamic acid led to increased dietary thiamine requirements [20]. The reason for this increased requirement is unknown. Conversely, dietary protein and fat have been found to reduce the metabolic demand for thiamine when compared to carbohydrates [91]. This is likely due to the role of thiamine as a cofactor in carbohydrate metabolism and the increased requirement for thiamine with activation of the Krebs cycle for the catabolism of dietary carbohydrates.

In adult humans, increasing one’s consumption of carbohydrates but maintaining a constant thiamine intake may lead to significant declines in plasma and urine thiamine content, but may not affect ETKA or fecal thiamine content [31]. Research determining the relationship between carbohydrate intake and thiamine requirements has not been conducted in dogs and cats. This is an area that warrants further research to better delineate thiamine requirements of complete and balanced commercial dog and cat foods that may contain varying carbohydrate concentrations.

e. Inadequate Food Intake

While insufficient dietary thiamine concentrations can be a risk factor for deficiency in dogs and cats ingesting their daily caloric requirements, decreased food intake (hyporexia) or complete lack of food intake (anorexia) can also increase the risk. In these cases, the daily recommended intake of thiamine that is necessary to meet the body’s consistent requirement for thiamine is not ingested.

f. Life Stage Requirements

Elderly individuals may be at higher risk of developing thiamine deficiency, as thiamine absorption is thought to become less efficient as humans age. Thiamine status using ETKA, tends to decline with age [18,22]. However, while analysis of RBCs by HPLC showed no difference between elderly and young adult groups [18], other research showed an age-related decline in TDP status [92]. Elderly individuals may have lower thiamine intakes than young adults, but intake may still meet nutrient recommendations for adult humans [18]. As few studies assessed dietary thiamine intake in relation to thiamine status, it is unclear if elderly humans have low thiamine status due to lower thiamine intake, as a result of age-related changes in absorption, or due to comorbidities. More research is therefore required to determine the effect of aging on thiamine metabolism in humans and even more so in pets, as currently no research is available investigating the effect of aging on thiamine status, metabolism and requirement of dogs and cats.

#### 4.2.2. Inadequate Thiamine Uptake

Dogs and cats may develop signs of thiamine deficiency despite ingesting nutritionally adequate concentrations of thiamine, which may be due to several factors affecting the uptake or metabolism of thiamine once it enters the body.

One such factor is intestinal malabsorption or maldigestion, which may occur with chronic GI diseases. More specifically, in humans, inflammatory bowel disease and gastric cancer have been found to be associated with symptoms of thiamine deficiency [93,94]. Thiamine deficiency can occur as a result of chronic vomiting as thiamine is unable to travel through the GI tract for absorption [95]. Also, chronic diarrhea can lead to poor intestinal uptake of thiamine.

Intestinal absorption of thiamine is regulated by two thiamine transporters (THTR), THTR-1 and THTR-2 [96]. Defects in these transporters may therefore be associated with thiamine deficiency. In cats, feline leukemia virus (FeLV), specifically the virus form known as subgroup A (FeLV-A), uses THTR-1 as a receptor to enter cells, which subsequently prevents thiamine uptake through these receptors [97,98]. However, due to THTR-2 not being affected by this mechanism, the risk of developing thiamine deficiency as a result of FeLV may be low. In dogs, Alaskan Husky Encephalopathy (AHE) is a generally fatal neurological disease that manifests in juvenile Alaskan Huskies, and the genomes of affected dogs have been found to contain a mutation in the gene that produces THTR-2, *SLC19A3* [99,100]. Dogs with AHE have a novel defect in one of two *SLC19A3* paralogs that has been found to lead to poor expression of this gene [19,100]. Clinical signs of this disease may therefore be related to thiamine deficiency of nervous tissues due to an inability to provide enough thiamine transporters to compensate for the low disease-related *SLC19A3* paralog expression [100].

Several disease states are also associated with thiamine deficiency. One such disease is congestive heart failure (CHF). A decreased thiamine status was found in humans with CHF, possibly as a result of diuretic-induced urine thiamin excretion, disease severity, malnutrition, and advanced age [32]. Individuals receiving thiamine via multivitamin supplementation were also less likely to have thiamine deficiency [32]. Cardiac failure has been experimentally induced in dogs given a thiamine-deficient diet [101], though some dogs in this study did not develop heart failure despite ingesting a thiamine-deficient diet. The diet that was fed was not complete and balanced and was potentially deficient in other nutrients as well, which may have confounded results. The relationship between CHF and thiamine deficiency in dogs and cats therefore requires further study. Moreover, in patients with CHF, the use of certain medications, such as diuretics, should also be considered when examining thiamine status.

#### 4.2.3. Excessive Thiamine Excretion

Because of the kidneys’ role in excretion and re-uptake of thiamine, diseases affecting the kidneys have an effect on thiamine status. In any disease associated with polyuria, the rate of urinary excretion is expected to be increased due to thiamine’s water-soluble nature. Research is scarce in dogs and cats. However, in humans, decreased renal function associated with type 1 and type 2 diabetes has been linked to thiamine deficiency due to excess excretion of thiamine compared to healthy individuals [43]. Moreover, in humans, a link also exists between chronic kidney disease (CKD) and thiamine deficiency [48]. Clinically, CKD is associated with polyuria and polydipsia in dogs and cats, and can also result in anorexia or hyporexia [102], all of which are risk factors of thiamine deficiency. Despite this, a recent study in dogs with CKD showed that these dogs had a higher blood TDP concentration compared to healthy controls [103]. The authors hypothesized that decreased renal function led to lower urinary thiamine excretion, though they did not assess rates of urinary thiamine excretion between dogs with CKD and controls. More research is therefore required to determine the effect of CKD on thiamine excretion.

Additionally, diuretics may increase the risk for thiamine deficiency due to the increased urinary flow rates associated with the use of diuretics. A study examining urinary thiamine excretion in rats compared different diuretics at increasing dosages, and found that furosemide and mannitol, as well as volume loading with isotonic saline solution, led to significantly higher urinary thiamine excretion compared to baseline values [104]. Increasing thiamine excretion was found to be related to increasing urine output, and urinary thiamine losses also tended to increase with increasing diuretic dosages. The authors reported that other diuretics tested in this study (chlorothiazide, acetazolamide, and amiloride) also showed higher urinary thiamine excretion compared to baseline, but detailed data for these medications were not provided and statistical significance was not reported.

### 4.3. Clinical Signs of Thiamine Deficiency

Signs of thiamine deficiency tend to be non-specific and can involve multiple organ systems [105], which can make the diagnosis challenging. In general, however, signs of thiamine deficiency in both dogs and cats are split into three progressive stages that are seen in both species: an induction stage, a critical stage, and a terminal stage [10].

The first stage, induction, occurs within one to two weeks of deficiency, and is characterized by a combination of vomiting, lethargy, and hyporexia or anorexia, though the animal’s behaviour may remain otherwise unchanged [4,28,106,107,108]. Few other signs occur at this stage. However, weight loss due to chronic poor food intake may be noted [6,28].

If deficiency is not reversed during the induction stage, the animal will enter the critical stage, and signs of nervous system damage will appear. In dogs and cats, these can include ataxia, paraparesis, nystagmus, delayed pupillary light response and blindness, recumbency, and increasingly poor proprioception, as well as seizures [4,5,9,28,35,38,106,109]. Cats may also exhibit cervical ventroflexion [106,109] and dyspnea [9,106]. Cardiovascular signs during chronic deficiency may include arrhythmias and bradycardia [110].

The terminal stage of thiamine deficiency begins after approximately a month of severe deficiency, and involves a rapid worsening of signs until death occurs. It is fatal within a few days unless supplementation occurs immediately [105]. Most cases of thiamine deficiency are diagnosed at the terminal stage [105].

### 4.4. Diagnosis of Thiamine Deficiency

Thiamine deficiency is poorly reflected on routine bloodwork. A complete blood count and serum biochemistry profile may be unremarkable in thiamine-deficient animals, even after prolonged deficiency [5,7,28,38,109]. High concentrations of organic acids that indicate impairment in thiamine metabolism may be used to diagnose thiamine deficiency [28,29]. In humans, hyperlactatemia has been noted as a possible symptom of thiamine deficiency, which does not respond to treatment with sodium bicarbonate and can be aggravated by administration of glucose [25,26]. Elevated lactate concentrations in urine may occur in dogs with thiamine deficiency [28,110], but these are not always present in cats [29]. As well, elevated lactate concentrations in plasma and cerebrospinal fluid (CSF) are not always present in dogs during thiamine deficiency [100], and CSF results can be unremarkable [7,106,109]. Blood pyruvate concentrations may be elevated in dogs with thiamine deficiency [110].

While several methods exist to assess thiamine status in blood, both directly and indirectly, analysis of thiamine status in veterinary patients is likely not regularly conducted, as thiamine status tends to not be reported in veterinary case reports pertaining to suspected thiamine deficiency. This may be due to the lack of an established reference range for thiamine status in dogs and cats, as well as poor availability of laboratories that routinely conduct thiamine analyses. Instead, thiamine deficiency in dogs and cats is typically diagnosed based on clinical signs and the presence of risk factors (e.g., unbalanced diet), and diagnosis is confirmed if signs reverse after thiamine supplementation [5,8,28,29,109,111,112]. An assessment of risk factors for thiamine deficiency should be accompanied by a nutritional assessment, such as the World Small Animal Veterinary Association (WSAVA) nutritional assessment guidelines, to rule out a nutritional cause [113]. Patients consuming raw or cooked homemade diets may be more likely to develop nutritional deficiencies due to consuming nutritionally unbalanced diets [50], which can include deficient thiamine intake. However, thiamine deficiency can also occur with commercial diets. Suspicion of a dietary cause of deficiency can be confirmed by analyzing a sample of the patient’s food for its total thiamine concentration.

Magnetic resonance imaging (MRI) can be used to reveal brain lesions commonly seen in thiamine deficiency. These tend to consist of symmetrical, bilateral lesions in the brainstem that primarily affect grey matter [29,114]. On histopathology, lesions are especially present in the nuclei of the caudal colliculi, and may either be spongy or involve cell degeneration and hypertrophy [4,5,9,29,38,114]. Lesions may also be present in the cerebral cortex, cerebellar noduli, rostral colliculi, and suprasplenial gyri of the caudal cortex, and the medial vestibular nuclei [4,5,108,109,114]. One report revealed unremarkable MRI findings despite presenting with clinical signs of thiamine deficiency in a cat, though it is unclear if signs reversed due to the cat having received supplemental thiamine for several days prior to MRI, or if no abnormalities had been present prior to supplementation [106].

### 4.5. Treatment of Thiamine Deficiency

Treatment of thiamine deficiency requires thiamine supplementation, either as a vitamin supplement or included in a complete and balanced diet. If thiamine deficiency is suspected due to an unbalanced diet, and if the suspected deficiency is discovered before the onset of advanced clinical signs, administration of supplemental thiamine and a change to a complete and balanced diet may be all that is required to prevent the appearance of further signs of deficiency [4,111].

As one of the signs of thiamine deficiency is anorexia [10], oral administration of thiamine as part of a complete and balanced commercial canned critical care diet or a veterinary liquid diet may require a feeding tube if the patient refuses or is unable to eat independently. Enteral feeding of patients with prolonged poor food intake should begin at one-third of the patient’s resting energy requirements [RER = 70 × (body weight _kg_^0.75^)], then increase to 2/3 and then to the patient’s full RER every 24 h or slower in order to avoid metabolic complications [115]. Veterinary liquid diets tend to contain adequate concentrations of thiamine. A recent study examined nutrient composition of seven veterinary liquid feline diets and found thiamine in sufficient concentrations in all but one analyzed diet [116]. Adequate thiamine supplementation is therefore expected in the majority of veterinary liquid diets. This suggests that supplying thiamine through a complete and balanced canned critical care diet or veterinary liquid diet may be a viable option for patients that are able to be fed enterally. An oral thiamine supplement may also be administered to patients; however, large oral doses of synthetic thiamine may be poorly absorbed in dogs [117], which would suggest that other forms of supplementation may be more effective.

Parenteral thiamine, typically in the form of thiamine hydrochloride, may be administered via intramuscular (IM), intravenous (IV), or subcutaneous (SQ) injections [105]. In the authors’ experience, dilution with sterile saline reduces potential injection-related discomfort in patients receiving SQ thiamine injections [40]. The ideal dose for supplemental thiamine is not currently known, and dosages reported in the literature for parenteral supplementation vary. In adult cats, dosages range from 20–300 mg per day [7,8,29,38,106,111], while in adult dogs they can range from 50–1250 mg per day [5,28,35]. Supplementation may begin with a loading dose of thiamine with subsequent smaller doses, or may utilize consistent dosages throughout treatment. Supplementation may occur as multiple doses per day, or may be administered once daily. However, dosages tend to be reported per animal rather than per kilogram, and the routes of thiamine administration vary between cases, therefore comparisons of precise dosages are difficult. There have been reports of severe reactions, such as anaphylaxis; neuromuscular, ganglionic, and vagus blockade; apnea; and hypotension associated with IV administration in dogs and cats [10,118], and it is therefore discouraged as a method of thiamine supplementation [105].

### 4.6. Prognosis and Outcome of Thiamine Deficiency

The severity of thiamine deficiency may have an impact on outcomes. To date, no research is available in dogs and cats. Nonetheless, a retrospective study of human hospital patients found that mortality rates were higher in human patients with severe thiamine deficiency compared to patients with slight or no deficiency [119]. However, due to the retrospective nature of this study, the researchers were unable to quantify illness severity or nutritional status in patients.

Signs of thiamine deficiency may begin to resolve within several hours after supplementation [5,29,107,111]. One case series found that 76% (13/17) of cats being fed a thiamine-deficient diet showed significant improvement within one week when treatment consisted of supplementation in addition to a diet change to a complete and balanced feline diet [106]. Resolution of MRI signs may be seen within days of beginning thiamine supplementation [8,28,106], but MRI abnormalities may persist for several weeks [28]. Additionally, clinical signs may remain despite absence of MRI signs [28,106]. Some neurological signs may persist for days to months after treatment ends [6,10,28,29,106], and neurological signs such as mild ataxia may persist for up to two years after treatment [106].

## 5. Conclusions

As thiamine is a dietary requirement in mammals, consistent and adequate daily intake is essential to prevent deficiency. This can be through a complete and balanced commercial diet, or a nutritionally balanced homemade diet formulated by a veterinary nutritionist to meet a dog or cat’s nutrient requirements for their respective life stage. Signs of deficiency in dogs and cats are non-specific in the induction stage, involve multiple organ systems, and diagnostic tests are not readily available, making early diagnosis of thiamine deficiency a challenge. As a result, most cases are diagnosed in the terminal stage, where quick identification based on clinical signs is critical and treatment must commence immediately to ensure the survival of the deficient dog or cat. The prevalence of thiamine deficiency in companion animals presenting to veterinary hospitals and ICUs is currently unknown. Parenteral thiamine is routinely administered in human hospitals for patients considered to be at high risk for deficiency [120], but the same is not necessarily true for dogs and cats presenting to veterinary hospitals. Thus, the need for routine thiamine administration in dogs and cats must be assessed in order to determine whether thiamine supplementation should be included as a routine part of medical care for dogs and cats presenting to veterinary ICUs.

## Figures and Tables

**Table 1:** Recommendations for dietary thiamine set by the NRC (2006), AAFCO (2017), and FEDIAF (2017) for adult dogs and cats.

Organization	Nutritional Basis	Species	Minimum Adequate Intake	Minimum Recommended Allowance
NRC [12]	DM Basis ^1^ (mg/kg DMB)	Cats	4.4	5.6
		Dogs	1.8	2.25
	Caloric Basis (mg/1000 kcal ME)	Cats	1.1	1.4
		Dogs	0.45	0.56
	Metabolic Body Weight	Cats ^2^ (mg/kg^0.67^)	0.11	0.14
		Dogs (mg/kg^0.75^)	0.059	0.074
AAFCO [13]	DM Basis ^1^ (mg/kg DMB)	Cats	--	5.6
		Dogs	--	2.25
	Caloric Basis (mg/1000 kcal ME)	Cats	--	1.4
		Dogs	--	0.56
FEDIAF [14]	DM Basis ^1^ (mg/kg DMB)	Cats	--	4.4 ^3^
				5.9 ^4^
		Dogs	--	2.1 ^5^
				2.5 ^6^
	Caloric Basis (mg/1000 kcal ME)	Cats	--	1.10 ^3^
				1.47 ^4^
		Dogs	--	0.54 ^5^
				0.62 ^6^

AAFCO—Association of American Feed Control Officials; FEDIAF—Fédération Européenne de l’Industrie des Aliments Pour Animaux Familiers (European Pet Food Industry Federation); NRC—National Research Council; DMB—dry matter basis; ME—metabolisable energy; ^1^ Based on a dietary energy density of 4000 kcal ME/kg; ^2^ Based on a lean cat with an energy intake of 100 kcal x BW^0.67^; ^3^ Based on a cat with a daily energy requirement of 100 kcal/kg^0.67^; ^4^ Based on a cat with a daily energy requirement of 75 kcal/kg^0.67^; ^5^ Based on a dog with a daily energy requirement of 110 kcal ME/kg^0.75^; ^6^ Based on a dog with a daily energy requirement of 95 kcal ME/kg^0.75^.
